# Supplementary material for: Minor changes in the incidence of primary and secondary involuntary childlessness across birth cohorts 1916 to 1975, but major differences in treatment success
Source: PLoS One. 2023 Jul 12;18(7):e0287540. doi: 10.1371/journal.pone.0287540 (PMC10337864; doi:10.1371/journal.pone.0287540)
Supplement: Attachment — Submitted filename: Response-to-reviewers-PONE-D-23-03364_R1.docx [file pone.0287540.s002.docx]

|  | **Avdeling / Institutt / Enhet**  Deres ref.:  Vår ref.:  Dato: June 5th, 2023  **Unntatt offentlighet jf: offl. § 25,1** |
| --- | --- |

**Responses to reviewers 2^nd^ revision**

**Reviewers' comments to first revision:**

Reviewer #2: All comments have been addressed

Reviewer #3: All comments have been addressed

2. Is the manuscript technically sound, and do the data support the conclusions?

Reviewer #2: Yes

Reviewer #3: Partly

3. Has the statistical analysis been performed appropriately and rigorously?

Reviewer #2: Yes

Reviewer #3: No

4. Have the authors made all data underlying the findings in their manuscript fully available?

Reviewer #2: Yes

Reviewer #3: Yes

5. Is the manuscript presented in an intelligible fashion and written in standard English?

Reviewer #2: Yes

Reviewer #3: No

6. Review Comments to the Author

Reviewer #2: The authors have been able to address all the issues I raised and corrected the typographical and grammatical errors.

Reviewer #3: Thank you for interesting paper. However:

1. Sampling method and sample size need to be Stated

Response: The first sentence of Material and Methods; page 4, lines 82-83 is rewritten to:

“The Tromsø Study (7, 8) is a large population-based survey that invites all Tromsø residents aged 40-98 years identified in the Central Population Register of Norway to participate in health surveys at timely intervals.”.

Sample size and response rates are displayed in 1^st^ paragraph of Results; page 6, starting at line 134.

2. I think this is retrospective cross sectional study design

Response: “Surveys” are by nature “cross-sectional studies”.

I have written “survey” in Material and Methods. In the discussion, page 11, line 206, I have changed the word “survey” with “cross-sectional study”. In other places in the discussion part I have kept the word “survey”.

3. Any ethical approval or waiver?

Response: Page 6, starting at line 129, the following statement explains “ethical status”:

“The Regional Committee for Medical and Health Research Ethics North (case no. 54329) and The Norwegian Centre for Research Data (project no. 298477) reviewed the protocol before study start. All participants gave written informed consent.”

4. English needs thorough editing because some sentences especially in the abstract are not making sense.

Response: An international (American) editing company has given the attached ms a final assessment.

Looking forward to a final decision!

Best wishes,

Finn Egil Skjeldestad, MD, PhD

Professor
